# Perceived health outcomes of recreation and happiness: exploring the mediating role of resilience

**DOI:** 10.3389/fpubh.2024.1383367

**Published:** 2024-09-11

**Authors:** Halil Sarol, Sezen Çimen Polat, Erdoğan Ekinci

**Affiliations:** ^1^Recreation Department, Faculty of Sport Sciences, Gazi University, Ankara, Türkiye; ^2^Trainer Education Department, Faculty of Sport Sciences, Gazi University, Ankara, Türkiye; ^3^Sports Management Department, Arhavi Vocational School, Artvin Çoruh University, Artvin, Türkiye

**Keywords:** leisure, perceived health, happiness, resilience, mediation effect

## Abstract

**Introduction:**

Previous research has shown that leisure activities can positively influence perceived health outcomes by increasing individuals’ levels of physical activity. Yet, little has been discovered about the mechanisms that are driving this association. This study was conducted with the aim of examining the relationship between perceived health outcomes of recreation and happiness as well as the mediating effect of resilience.

**Methods:**

The study comprised a total of 451 adult individuals residing in seven different geographical regions of Turkey, who were included using a convenience sampling method, and the data were collected between March and June 2023. Respondents completed assessments utilizing the Perceived Health Outcomes of Recreation, Brief Resilience Scale, and The Oxford Happiness Questionnaire-Short Form. Data analysis was executed employing the PROCESS macro for SPSS.

**Results:**

The study’s findings revealed that perceived health outcomes of recreation had a positive effect on both happiness (β = 0.146, [95% CI: 0.106, 0.186]) and resilience (β = 0.156, [95% CI: 0.107, 0.205]). Resilience had a positive effect on happiness (β = 0.435, [95% CI: 0.362, 0.507]). Furthermore, the relationship between perceived health outcomes of recreation and happiness was partially mediated by resilience (β = 0.068, [95% CI: 0.042, 0.096]).

**Conclusion:**

As a result, participating in leisure activities has been found to have a positive impact on perceived health outcomes, which in turn positively affects both happiness and resilience. Additionally, psychological resilience can be said to partially mediate the relationship between perceived health outcomes of recreation and happiness.

## 1 Introduction

The inception of the “Benefits Movement,” spurred by the growing imperative to assess and communicate the advantages of recreational activities to the public, can be traced back to the 1990s (1). This movement has systematically presented the impacts of leisure-time pursuits on both physical and psychological well-being, substantiated by empirical evidence (2, 3). Within this framework, the contemporary issue of a sedentary lifestyle has been underscored, highlighting the need to advocate for increased physical activity in societies (4, 5). Numerous studies have found a reduction in adverse psychological symptoms, such as depression and anxiety, alongside an elevation in psychological well-being and life satisfaction among individuals engaged in physical activity (6–11).

However, since 2019, the social isolation measures and lifestyle alterations resulting from the global impact of the COVID-19 pandemic have led to heightened sedentary behaviors that adversely affect mental health (12, 13). Research conducted during the pandemic has proposed that even participating in low-intensity physical activities could contribute to the reduction of stress and anxiety levels among individuals (14, 15). Furthermore, engaging in both outdoor and home-based leisure activities during the pandemic has emerged as a robust indicator of increased happiness, life satisfaction, and health perception, particularly among adults (16). The emphasis on physical activity as a means to enhance immunity, preserve cardiovascular health, and improve mental well-being has gained prominence after the pandemic, supported by various studies (17–20). In essence, pivotal aspects such as perceived health, happiness, and psychological resilience in recreation warrant further exploration and in-depth analysis in the relevant literature after the COVID-19 pandemic.

### 1.1 Background literature and hypotheses

#### 1.1.1 Perceived health outcomes of recreation and happiness

The term “perceived health outcomes of recreation” refers to the influence of engagement in leisure activities on emotional, environmental, intellectual, social, spiritual, and physical health (21, 22). Additionally, perceived health outcomes of recreation encompass goal orientation, positive emotions, overcoming negative emotions, promoting relationships, and fostering tranquility (23). In this context, the positive evaluation of significant aspects of one’s life or an indirect judgment that life is going well, known as the concept of happiness (24), is believed to be connected to perceived health in recreation. Studies suggest that fulfilling recreation experiences have the potential to positively impact subjective well-being and mental health (25, 26). A study by Temel and Tükel (27) identified a significant and positive relationship between perceived health outcomes and happiness. Another study found a positive association between participation in outdoor and home-based activities and happiness and health perception among adults (28). These findings suggest that engaging in recreational activities may have positive effects on adults’ perceived health outcomes, thereby contributing to their happiness and overall well-being.

#### 1.1.2 Mediating role of resilience

Psychological resilience, characterized by flexibility and quick recovery, has been the focus of numerous studies that have explored its enhancement through leisure activities (29–32). These studies indicated that psychologically resilient individuals actively employ leisure-based coping strategies to cultivate positive emotions and enhance well-being.

**Figure 1:**
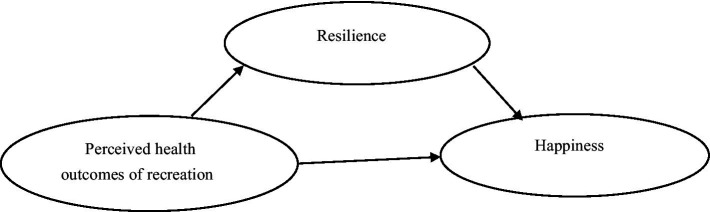
Proposed mediating model.

Fredrickson’s (33) broaden-and-build theory posits that positive emotions play a crucial role in expanding an individual’s momentary thought-action repertoire, which in turn builds their personal resources over time. This theory emphasizes that positive emotions, such as happiness, not only make individuals feel good in the moment but also provide long-term benefits for their psychological and physical health. In this context, according to Fredrickson’s broaden-and-build theory, leisure activities can be leveraged not only to experience momentary happiness but also to build resilience and promote long-term well-being (29, 30). Additionally, it is suggested that socializing through leisure activities contributes to the development of psychological resilience (32). Moreover, engaging in physical activity is known to assist in the enhancement of psychological resilience (34). Another study found that individuals with higher mental resilience scores tend to have better perceived health outcomes (35). Based on this evidence, it is assumed that psychological resilience is positively associated with perceived health outcomes in recreation. Consequently, a study model has been developed to evaluate the following hypotheses (Figure 1).

*H1:* Perceived health outcomes of recreation are positively correlated with resilience.
*H2:* Perceived health outcomes of recreation are positively correlated with happiness.
*H3:* Resilience is positively correlated with happiness.
*H4:* Resilience plays a mediating role between perceived health outcomes of recreation and happiness.

## 2 Methods

### 2.1 Sample and procedure

The study group comprised 451 adult individuals, with 271 (60.1%) men and 180 (39.9%) women residing in seven geographical regions of Türkiye. The participants, aged between 18 and 71 years (average age = 34.73 years), were recruited through convenience sampling. The males in the study had a mean age (M) of 35.89 years (SD = 12.38), while the females had a mean age of 32.99 years (SD = 10.76). In this study, participants were asked about the frequency with which they engaged in a wide range of leisure activities, such as walking, jogging, cycling, and swimming, all considered physical exercise. Concerning physical activity participation, 19.5% reported rare engagement, 37.3% occasional, and 43.2% regular participation. Individuals who did not participate in any physical activity in their leisure time were not included in the study.

The study survey, conducted online, was disseminated through social media platforms including Facebook pages, WhatsApp groups and Twitter throughout November and December 2023. Participants were provided with comprehensive written information about the research objectives, scientific content, and the anonymous nature of the survey. Participants in the study had to be no younger than 18 years old, free of any health conditions that might prevent them from participating in physical activity, and sign an informed consent form. These inclusion criteria ensured that subjects could provide informed and voluntary consent to the study. Participants reported spending an average of 8–10 min completing the research form. To maintain data integrity, the survey required responses to all questions, and incomplete survey forms were excluded from the analysis. Out of a total of 473 respondents, data from 451 participants were included in the study after excluding 22 individuals who did not meet the eligibility criteria, and outliers were removed during this process.

### 2.2 Measures

The data collection tool used in this study comprised two main sections. The first section gathered information on participant demographics and physical activity engagement. Physical activity was measured using a questionnaire designed by the researchers. Participants were asked to report the frequency of their physical activity in the past week (rarely, occasionally, and regularly). The second section incorporated the following measurement tools.

#### 2.2.1 Perceived health outcomes of recreation scale

Developed by Gómez et al. (21) and adapted to Turkish by Lapa et al. (55), PHORS consists of 16 items (e.g., causes me to enjoy life more and reduces my chance of weight gain) organized into three subdimensions: “improved condition,” “prevention of worse condition,” and “realization of a psychological experience.” Participants rated each item on a seven-point Likert scale, ranging from 1 (never like me) to 7 (very much like me). The Cronbach’s alpha reliability coefficient was calculated and found to be 0.97.

#### 2.2.2 The brief resilience scale

Developed by Smith et al. (56) to measure individual resilience, the Turkish validity and reliability study of BRS was conducted by Doğan (57). The scale has a single-dimensional structure with six items (e.g., I tend to bounce back quickly after hard times), wherein items 2, 4, and 6 are negatively worded. All items were rated on a five-point Likert scale ranging from 1 (strongly disagree) to 5 (strongly agree). In the scope of this study, Cronbach’s alpha coefficient was calculated to be 0.80, indicating a high level of internal consistency.

#### 2.2.3 The oxford happiness questionnaire-short form

Adapted to Turkish culture by Doğan and Çötok (58), this scale was developed by Hills and Argyle (59). The scale includes seven items, with items 1 and 7 reverse-scored. Participants evaluated items on a Likert-type scale ranging from 1 (strongly disagree) to 5 (strongly agree). The Cronbach’s alpha internal consistency coefficient was calculated for the scale within the scope of this study and was found to be 0.80.

### 2.3 Data analysis

In this study, the structural equation model was employed to investigate the influence of perceived health outcomes of recreation on happiness mediated by psychological resilience. Data analysis was conducted using SPSS 23.00 software. Initial data scrutiny involved the identification and handling of missing or erroneous values, and outliers were excluded. The normality of the data was assessed through skewness and kurtosis coefficients, with a range of −1 to +1 considered indicative of normal distribution (36). Bivariate outliers were identified using Mahalanobis distance analysis, which assesses how far each data point is from the mean of the multivariate distribution (37). Additionally, a scatterplot was examined to observe points that deviate significantly from the general trend or differ markedly from other points. It has been established that there were no bivariate outliers in the dataset used for the analyses, ensuring that they do not impede the mediation analysis.

Basic descriptive statistics were calculated, and Pearson Product–Moment Correlation analysis was performed to examine the interrelationships between the study variables. Finally, the mediating role of resilience in the relationship between perceived health outcomes of recreation and happiness was explored using the PROCESS macro (Model 4) for IBM SPSS Statics (Version 27) (38), following methodology of Hayes (39). The analysis incorporated 5,000 bootstrapped samples, and 95% bias-corrected confidence intervals were used to assess the statistical significance of mediated models and the magnitude of the mediated effect. In mediation analysis, the literature suggests varying sample size requirements between partial and complete mediation analyses, with partial mediation typically requiring a minimum of 340 participants. Our study included 451 participants and utilized the bootstrapping method for analysis, ensuring sufficient sample size for robust statistical inference (40).

## 3 Results

### 3.1 Descriptive statistics

Descriptive statistics and the correlation matrix for the variables in the model—perceived health outcomes of recreation (independent variable), happiness (dependent variable), and resilience (mediating variable)—were assessed via correlation analysis. The results are presented in Table 1.

**Table 1:** Demographic information.

		*N*	%
Gender	Women	180	60.1
	Men	271	39.9
Age	18–27	162	35.9
	28–37	112	24.8
	38–47	102	22.6
	47 and above	75	16.6
Marital status	Single	235	52.1
	Married	216	47.9
Physical activity participation frequency	Rare	88	19.5
	Occasional (1–2 day per week)	168	37.3
	Regular (3 and above per week)	195	43.2

The analysis revealed a statistically significant and positive relationship between perceived health outcomes of recreation and resilience (*r* = 0.28, *p* = 0.01), as well as happiness (*r* = 0.41, *p* = 0.01). Similarly, a statistically significant and positive relationship was observed between the mediating variable of resilience and happiness (*r* = 0.54, *p* = 0.01). Skewness and kurtosis values for PHORS (skewness = −1.59, kurtosis = 2.51, SD = 0.22), resilience (skewness = 0.51, kurtosis = 0.15), and happiness (skewness = −0.16, kurtosis = 2.51, SD = −0.10) were within acceptable ranges (−3 to +10) (41). Additionally, the Cronbach’s alpha internal consistency values for the Perceived Health Outcomes of Recreation Scale (α = 0.97), Brief Resilience Scale (α = 0.81), and The Oxford Happiness Questionnaire (α = 0.80) were within acceptable limits (60).

The PROCESS macro analysis results (Table 2) indicated that perceived health outcomes of recreation (β = 0.1461, *p* < 0.001, *t* = 7.1662, [CI = 0.1061, 0.1862]) and resilience (β = 0.1565, *p* < 0.001, *t* = 6.2282, [CI = 0.1071, 0.2059]) significantly and positively influenced happiness. Furthermore, the indirect effect of perceived health outcomes of recreation on happiness through resilience was significant, suggesting that resilience partially mediates the relationship between perceived health outcomes of recreation and happiness (β = 0.0681, *p* < 0.001, SE = 0.0138, [CI = 0.0429, 0.0967]). According to the analysis results presented in Table 2, after the mediator variable was entered into the model, its effect on perceived health outcomes of recreation happiness decreased (from 0.14 to 0.06). The completely standardized effect size of the mediation effect was 0.1308, suggesting a moderate effect size given that values around 0.09 are considered partially moderate (42). All coefficients presented in Table 2 are standardized beta coefficients. Conclusions based on confidence intervals related to these standardized coefficients are delineated in the table. Based on these results, it can be concluded that the study hypotheses were supported (Table 3).

**Table 2:** Descriptive statistics and correlation matrix of all variables.

	M ± SD	1	2	3
1. Perceived health outcomes of recreation scale	5.79 ± 1.33	-		
2. Brief resilience scale	3.41 ± 0.74	0.28**	-	
3. The Oxford happiness questionnaire-short form	3.57 ± 0.69	0.41**	0.54**	-

*N* = 451. **p < 0.01.

**Table 3:** Mediating effect of resilience on the relationship between perceived health outcomes of recreation and happiness.

Effects	Standardized			
	β	SE	LLCI	ULLC
→ PHORS OHQ-SF	0.1461**	0.0204	0.1061	0.1862
→ PHORS BRS	0.1565**	0.0251	0.1071	0.2059
→ BRS OHQ-SF	0.4350**	0.0367	0.3628	0.5072
→ → Indirect effects PHORS BRS OHQ	0.0681	0.0138	0.0429	0.0967

*N* = 451, **p < 0.01. PHORS, Perceived health outcomes of recreation scale; BRS, Brief resilience scale; OHQ-SF, Oxford happiness questionnaire-short form.

## 4 Discussion

In various studies dedicated to the exploration of physical activity in leisure time, researchers have individually investigated perceived health outcomes (16, 43), happiness (44), and psychological resilience (45). However, an apparent gap exists in the literature regarding examining interconnections between these three variables. Thus, the primary objective of this study is to delve into the mediating role of psychological resilience in the relationship between perceived health outcomes in recreation and happiness.

In line with the first hypothesis of our study, the findings reveal significant and positive relationships between perceived health outcomes of recreation and psychological resilience. The standardized regression coefficients indicate a direct and substantial impact of perceived health outcomes of recreation on psychological resilience. While few studies have specifically explored the connection between perceived health outcomes of recreation and psychological resilience in the existing literature, it is noteworthy that various studies have investigated the relationship between perceived health outcomes and psychological resilience, incorporating both concepts into structural models (35, 46, 47). Despite variations in sample groups across these studies, a consistent relationship between perceived health outcomes of recreation and psychological resilience is discernible.

In line with the second hypothesis of the study, a significant relationship was found between perceived health outcomes of recreation and happiness. Based on the research findings, there exists a statistically significant positive correlation between perceived health outcomes of recreation and happiness scores. A comprehensive review of the literature further supports these research findings, with numerous studies aligning with the observed correlation (27, 28, 48). For instance, a study focusing on outdoor adventure recreation activities found that participation in such activities contributes to improved physical and psychological health outcomes, thereby enhancing overall happiness (16). In light of these findings, it can be reasonably asserted that engagement in recreational activities may positively impact happiness through the perceived health outcomes associated with those activities.

In accordance with the third hypothesis of the study, the results indicate significant relationships between happiness and resilience. The analysis within our model reveals that psychological resilience exerts a direct and meaningful impact on happiness. This implies that as participants’ levels of psychological resilience increase, their perceptions of happiness also increase. Consistent findings have been reported in previous studies that specifically investigated the relationship between happiness and psychological resilience (49–51). These findings underscore the pivotal role of resilience as a factor contributing to the promotion of happiness and overall well-being. Furthermore, it can be asserted that increased levels of resilience are associated with higher levels of happiness.

According to the final hypothesis in our study, resilience plays a mediating role between perceived health outcomes of recreation and happiness. The results indicate that the perceived health benefits of recreation also increase psychological resilience. In this context, individuals become more resilient as they feel healthier and more capable due to recreational activities. This increased resilience contributes to higher levels of happiness. In short, psychological resilience helps individual’s better cope with stress and challenges and maintain a positive outlook on life, which increases their overall happiness. Furthermore, a significant body of literature demonstrates the mediating effect of resilience in various contexts, such as the relationship between physical activity and mental health (30, 32, 52). Numerous studies have investigated constructs similar to those examined in this research (self-esteem, self-efficacy, and stress). Although the present study focuses on different variables, it allows us to establish connections between our findings and previous research. However, drawing from prior research (29, 30, 32, 53, 54) and the current findings, it can be inferred that psychological resilience exerts a partially mediating effect on the impact of perceived health outcomes of recreation on happiness. This suggests that the indirect assessment of life well-being, influenced by the effects of leisure participation on emotional, environmental, intellectual, social, spiritual, and physical health, may vary depending on an individual’s level of psychological resilience. These findings underscore the importance of considering factors beyond direct measures of well-being when evaluating an individual’s overall life satisfaction and fulfillment. It can be argued that individuals with higher psychological resilience derive greater benefits from engaging in leisure activities, leading to improved perceived health outcomes. Psychological resilience plays a critical role in facilitating the process by which recreational activities buffer stress, enhance coping skills, foster positive emotions, and promote happiness. Moreover, the extent to which leisure activities affect these indirect aspects of an individual’s well-being may vary according to their level of psychological resilience. In other words, people who are more psychologically resilient may gain greater benefits from participating in leisure activities. Their resilience can help them cope better with challenges and stressors, thereby enhancing their emotional, social, and physical well-being influenced by leisure pursuits. Conversely, individuals with lower levels of psychological resilience may not reap the same degree of indirect benefits from leisure participation. Their ability to achieve positive impacts on various dimensions of well-being through leisure activities might be limited by their level of resilience. Thus, the impact of leisure participation on overall life well-being is not uniform across individuals and can be influenced by their psychological resilience. Recognizing this variability is crucial for understanding how leisure activities contribute to people’s overall quality of life.

## 5 Conclusion

This study aims to investigate the mediating role of psychological resilience in the relationship between perceived health outcomes of recreation and happiness. Research shows that the positive impact of leisure activities on happiness is not just a direct relationship. The relationship between perceived health outcomes of recreation and happiness is partially mediated by resilience, highlighting the importance of psychological factors in the well-being derived from leisure activities. Moreover, it is partly explained by the increase in psychological resilience that these activities stimulate. In this context, while recreation directly increases happiness, it also makes people more psychologically resilient, which further increases their happiness. Research highlights the importance of encouraging recreational activities as a way to improve both physical and mental health. Encouraging regular participation in recreational activities may be a practical approach to increasing individuals’ happiness and overall life satisfaction. Developing community-based recreation programs can help individuals increase their resilience and therefore their ability to cope with life’s challenges. Policies that support work-life balance and allow time for recreational activities can contribute to the well-being of the population. Policy practitioners will achieve significant results when public health and well-being programs include strategies that support resilience. At this point, policy implementers should consider investing in recreation infrastructure and creating accessible opportunities for people to participate in leisure activities.

## 6 Limitations

Although our study offers insights into how adult participants perceive the health benefits of recreation and happiness, it has certain limitations. First, self-reported data used in our study could have introduced response bias. Second, the cross-sectional design of the study makes it more difficult to determine causality, emphasizing the necessity of longitudinal studies. In addition, because the survey data were collected online, there is a risk of over-representation of those likely to participate in online surveys, and under-representation of some demographic groups. Furthermore, future research should use qualitative investigations to acquire comprehensive understanding of the complex experiences and viewpoints involved in the interaction between adult resilience, happiness, and recreation.

## Data Availability

The original contributions presented in the study are included in the article/supplementary material; further inquiries can be directed to the corresponding author.
